# Colouring Flowers: Books, Art, and Experiment in the Household of Margery and Henry Power

**DOI:** 10.1017/S0007087422000474

**Published:** 2023-01-13

## Abstract

This article examines the early-modern household’s importance for producing experimental knowledge through the Halifax household of Margery and Henry Power. While Henry Power has been studied as a natural philosopher within the male-dominated intellectual circles of Cambridge and London, the epistemic labour of his wife, Margery Power, has hitherto been overlooked. Since the 1650s, this couple worked in tandem to enhance their understanding of the vegetable world through various paper technologies, from books, paper slips, and recipe notebooks to Margery’s drawing album and Henry’s published *Experimental Philosophy*. Focusing on Margery’s practice of hand-colouring flower books, her copied and original drawings of flowers, and her experimental production of ink, we argue that Margery’s sensibility towards colour was crucial to Henry’s microscopic observations of plants. Even if Margery’s sophisticated knowledge of plants never left the household, we argue that her contribution was nevertheless crucial to the observation and representation of plants within the community of experimental philosophy. In this way, our article highlights the importance of female artists within the history of scientific observation, the use of books and paperwork in the botanical disciplines, and the relationship between household science and experimental philosophy.

In December 1668, Henry Power, Halifax physician, natural philosopher and one of the first elected members of the Royal Society, passed away in the city of Wakefield. A pioneer of the budding discipline of microscopy, Henry was known for his *Experimental Philosophy* (1664), the first English book on microscopical observations.^i^ However, it is less known that a woman was also involved in Henry’s microscopical observations of plants — his wife, Margery Power. Henry’s memorandum books show that Margery was likely to be in charge of purchasing and handling the scientific instruments at home, at least towards the end of Henry’s life. In October or November 1668, she noted down the purchase of ‘A Telescope’ for £4 and ‘2 Prospectifs’ for 14d, and on 5 April 1669, just after Henry had died, she wrote down a memo to ‘Remember to aske for the Mycroscope at Mr. Mat: Widdops’ with her signature ‘Margery Power’ above it.^ii^ While Samuel Pepys and his wife famously observed through a microscope together using Henry’s book as instruction, it seems that Margery also observed through microscopes, both together with Henry and by herself.^iii^

Brought up in the family of Anthony Foxcroft, a wealthy merchant family in the Halifax region just like the Power family, Margery had been receiving education and was familiar with the Halifax intellectuals in her own and the Power family’s circle. Around the time she married Henry, her father married Henry’s widowed mother, making Henry and Margery stepsiblings as well as husband and wife. According to Kim Sloan, Margery possibly took drawing lessons with her sister-in-law Ellen Power from the glass painter Henry Gyles.^iv^ The same day when she asked for the microscope, Margery made a note to ‘enquire of Mr Giles about lackering of Picture Frames & write down the Receipt’. All these notes indicate Margery’s knowledge and dedication to drawing.

Margery Power tells us a new story about the role and agency of female members of a scientific household. As Deborah Harkness has shown in her work on Jane and John Dee, the household of an early modern natural philosopher was often an “experimental household.”^v^ In this paper, we argue that housewifery tasks and everyday practices had not only logistic and practical importance, but also epistemic importance for the emerging culture of experimental philosophy, as the female members of the household also contributed intellectual labour such as identification of plants, flower colouring and painting, and experiments on the nature of colour. Using a painting kit Henry bought her, Margery hand-coloured numerous printed images of flowers in herbals and florilegium books. Both she and Ellen each produced a striking watercolour album in which they limned and coloured flowers, insects and animals following the styles of well-known botanical authors and illustrators such as Jacques Le Moyne de Morgues, Johann Theodor de Bry and John Parkinson. For at least 15 years, painting and colouring flowers was an integral part of Margery’s life, and a crucial factor for the development of experimental philosophy and microscopic vision within the Power household.

Drawing and colouring plants require a significant amount of botanical knowledge. To produce botanical drawings in correspondence to the plants she drew after, Margery read and analysed several herbals. In many cases, she compared botanical descriptions from several sources only to confirm the colour of one single species. She also conducted colourmaking experiments alongside Henry. Similarly, the carefully taken notes, comments and scribbles left by Margery on her husband’s herbal books and botanical notebooks all suggest the shared ownership and use of botanical knowledge within this house: the library, the plant samples, as well as the magnifying glasses and microscopes were never solely Henry Power’s tools for knowledge production.

Despite her thoughtful production of botanical drawings, Margery Power has not so far held a place in our histories of the arts and sciences. Margery never published any works of her own, and her scattered botanical notes and recipes could easily be undervalued within the traditional hierarchy of knowledge production: Authors prioritised over illustrators, ‘original’ work over commonplaces, and natural-historical observations carried out in scientific settings over household, ‘amateur’ flower painting. However, in recent years such hierarchies have been increasingly upset by scholarship within the history of art and science. Scholars like Alix Cooper, Mary Terrall and Anna Marie Roos have studied the critical role of sometimes named, sometimes unnamed female illustrators such as Hélène Dumoustier and Susanna and Anna Lister, and shown how they fostered what Lorraine Daston and Peter Galison have called a collaborative “four-eyed sight” of nature.^vi^ At the same time, Florike Egmond, Sachiko Kusukawa and others have argued that the practice of copying images was not only a way of learning to draw, but an integral part of the creation of knowledge that served various epistemic ends.^vii^

Yet, the epistemic importance of copied images speaks not only to images of Thomas Willis’s dissected brain or Robert Hooke’s enlarged snowflakes, but also to the feminised image reproduction of flower books, usually associated with embroidery and needlework. To reevaluate the epistemic importance of these traditionally feminine image genres, scholars such as Janice Neri and Mary Learner have pointed out that some natural history albums, like those of Maria Sibylla Merian, were specifically published to serve as inspiration for embroiderers, and that women embroiderers of botanical images shaped the vision promoted by male experimental philosophers.^viii^ Margery and Ellen’s drawings, nonetheless, were also copied from Jacques Le Moyne’s *La Clef des Champs*, a book recommended by the author as a pattern book for painters, engravers, embroiderers, weavers and needleworkers.^ix^ Building on this scholarship, we argue that the drawings of Margery and Ellen significantly shaped the vision of colours in Henry’s microscopical observations, which emphasizes the usually ignored household context of early modern microscopic practice. Whereas the few studies of Henry Power has mainly focused on his relation to the male-dominated intellectual spheres of Cambridge, the Royal Society and the so-called Towneley circle, we argue that Henry Power’s natural-historical vision was shaped by his collaboration with the female members of his household, Margery and Ellen Power.

Unlike in the collaboration between Susanna and Anna Lister and their father, the natural historian Martin Lister, Margery did not produce images that were published or in other ways used as natural-historical illustrations. Margery’s significance for early modern microscopy was not in published images, but in the sensibility of colours she created during in her process of image-making and colour-making experimentations. Margery’s paintings never left the house, but her knowledge of pigments, tones and hues significantly shaped Henry’s way of seeing and recognizing colour. As Henry confessed himself, compared to the shape and size, the exact colours of small microscopic specimens were harder to get hold of. Therefore, it was important for microscopists to master a sharp sense of colours and a refined chromatic vocabulary. In the scholarship on early modern microscopy emphasis is often put on Hooke’s circle of miniature painters and the training he received in the workshop of the portrait painter Peter Lely.^x^ It was his experience of collaborating with skilled artists that allowed Hooke to produce the magnificent illustrations of the *Micrographia*, and more importantly, sharpened his eye and allowed him to see the details he represented through text and image. In a similar manner, we argue that the household knowledge of producing, observing and representing colours pioneered by Margery shaped Henry’s description of the chromatic details of minute plant parts observed through his microscopes. While Brian Ogilvie makes a clear distinction between the technical language of botanical texts and the sparse verbal descriptions of the *florilegium* genre, Margery’s work shows that flower colouring could be an equally technical process. As we argue, the female amateur artist Margery produced a tested, recorded and regularised colour system that had a lasting impact on early modern microscopy.^xi^ From books and plants to microscopes, the case of Margery Power calls for a revaluation of the importance of female artists within the history of scientific observation, the use of books and paperwork in the botanical disciplines, and the relationship between household science and the emerging experimental philosophy.

## A Library of Their Own

**Fig.1:**
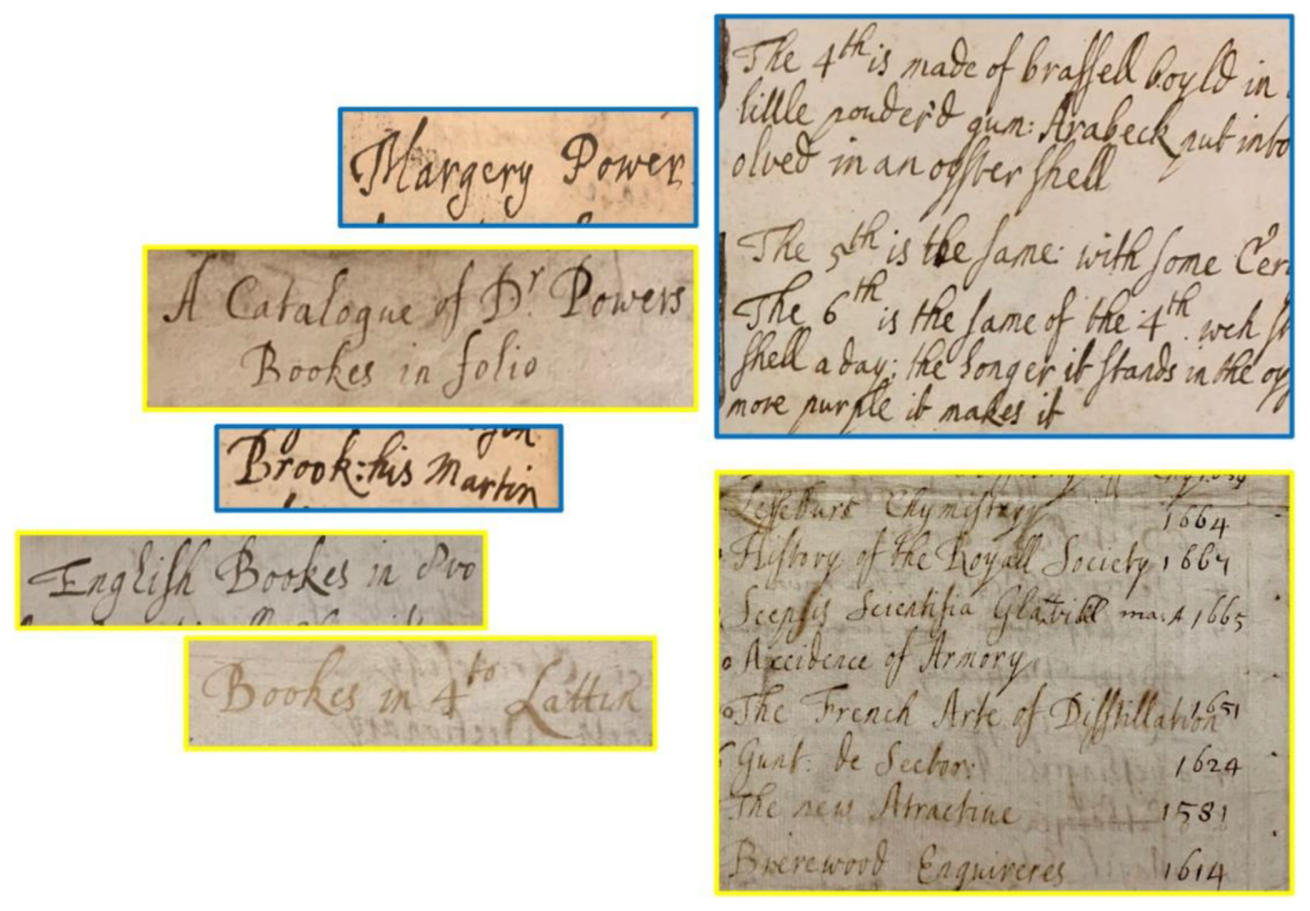
Comparison of Margery Power’s handwriting and the handwriting in Henry Power’s book catalogue in Sloane MS 4019, British Library. Images in blue frames were already identified as Margery’s handwriting, and images in yellow frames were handwriting in Sloane MS 4019 catalogue. Power: Sloane MS 4019, f.149; Sloane MS 1358, f.58, bills and notes written by Margery. Booke/Brooke: Sloane MS 4019, f.149, 152; Sloane MS 1358, f.65. General Comparison: Sloane MS 4019, f.150; Add MS 5298, f.1.

Henry Power cared about his books. Two months before his graduation day, the soon-to-be doctor took a good look around the study in his Halifax home, took out a brand new notebook, and started writing on the first page: “Left at Halifax May 4th 1654 when I went to Cambridge. On the high shelf — 37 Books in 4to. On the lowe shelfe 38 Bookes in 8vo & 4to togeather. 56 Bookes more in 8vo which lyes upon 13 thin folios on the lower shelfe. In the window 19 folio.”^xii^ 10 years later in 1664, preparing now to move his family from Halifax to Wakefield, Henry did the same thing once again. In the manuscript “A Catalogue of all my Bookes,” Henry wrote down the titles of all his books organized by language and size. During these 10 years, his book collection had vastly expanded: The number of folio books had increased from 32 to 68, the quartos to 74, and the octavos had increased to more than two hundred.^xiii^ When Henry died four years later in 1668, his wife Margery seemed to have inherited his books. ^xiv^ A library catalogue titled “A Catalogue of Dr Power’s Bookes” currently collected in Sloane MS 4019, f. 149-154, records the changes and expansions of this library. Written in a neat and legible hand, this catalogue noted down the authors and titles of the books more carefully, as well as the prices of all the books.^xv^ A comparison between Margery’s hand from her drawing album and other sources and the catalogue has shown that f. 149-153 of the catalogue were very likely to be written by Margery. (Fig.1) The book titles on the final two pages of this catalogue starting with “Latin Bookes in 12o” were written in another hand, potentially other family members or servants working with her, while the price and publication year were written consistently in Margery’s hand throughout the catalogue. As such, it seems probable that Margery, the widow, prepared this catalogue either to sell the books or to testify to their value.^xvi^

As Anthony Grafton summarised in 2006, “A historian of ideas working on a sixteenth- or a nineteenth-century thinker is likely to start, now, by asking how many books survive from the individual’s library and investigating notebooks to see how he or she processed what she read.”^xvii^ Today, this investigation has been expanded to thinkers of all genders who owned and read books in various ways, and in recent years, we have witnessed a growing interest in book ownership of learned women.^xviii^ The introduction of the perspective of gender in library studies requires us to reconsider private libraries that were traditionally considered owned by learned men. In the case of “Dr Power’s Bookes,” we argue that this library should be regarded as a shared household library used by both male and female members of the Power family. The fact that Margery was able to create an easily readable catalogue of these hundreds of books written in English, Latin, Spanish and French speaks to her familiarity with the library: based on Henry’s old catalogue, she added more bibliographical information such as author names and publication year into the new catalogue, and recorded titles of books they acquired after 1664. More importantly, this library does contain a book owned by Margery: in the 1664 catalogue, Henry noted down the existence of an unspecified folio book, which he catalogued as “my wifes Booke of Plants in Colours.”^xix^

This “my wifes Booke” could very possibly be a 1612 copy of Johann Theodor de Bry’s *Florilegium novum*: a folio flower book, hand-coloured by Margery herself with both oil and watercolour and catalogued in Sloane’s library as “painted over by Mrs Power” and in another entry “colour’d by Mrs Power.” ^xx^ It is the only book we know of with Margery Power’s signature on it. To be precise, it is a ‘compiled book’, not a normal book. While the frontispiece and foreword were from the 1612 edition, plates from later editions, including the 1613 and 1614 ones, were assembled into this one volume. The pagination was thus messy and no longer followed the sequence of the 1612 edition: Images of tulips, for example, have been moved from the beginning of the book to the end of the book. In some cases, there are even traces that the page numbers on the original plates were erased by some means.^xxi^ In this *Florilegium*, flowers on almost every page have been coloured by Margery, with delicate shades and diverse changing colours. She creatively used a combination of differently saturated yellow, green, greenish blue and shiny blackened oil paint to depict the withered parts of the leaves in contrast to the watercoloured fresh green leaves, which added to the three-dimensional depth of her colouring.

Although the *Florilegium* might be the only book Margery “owned,” it was far from the only book she worked with. Another book we know that Margery extensively coloured, a 1591 edition of Matthias de l’Obel’s *Icones Stirpium*, was nonetheless signed with Henry Power’s name. It was an expensive book of more than 800 pages that Henry bought for 14s., which could very possibly refer to the entry “(price) 14-0. An Old Lattin Herball, 1591” that appears in Margery’s catalogue of the library.^xxii^ It has Henry’s signatures all over the place: Two on the frontispiece, one under the preface title, some at the end of his annotations, and once he even added “H. ower” to the book’s signature “P” at the bottom.^xxiii^ Henry signed off with his title M.D. in this book, which indicates that this book was bought after his graduation in 1654 and possibly after his marriage. Margery Power did not sign Icones Stirpium, but the particular manner of colouring and inserting paper slips in her hand indicate her work on it.

**Fig.2:**
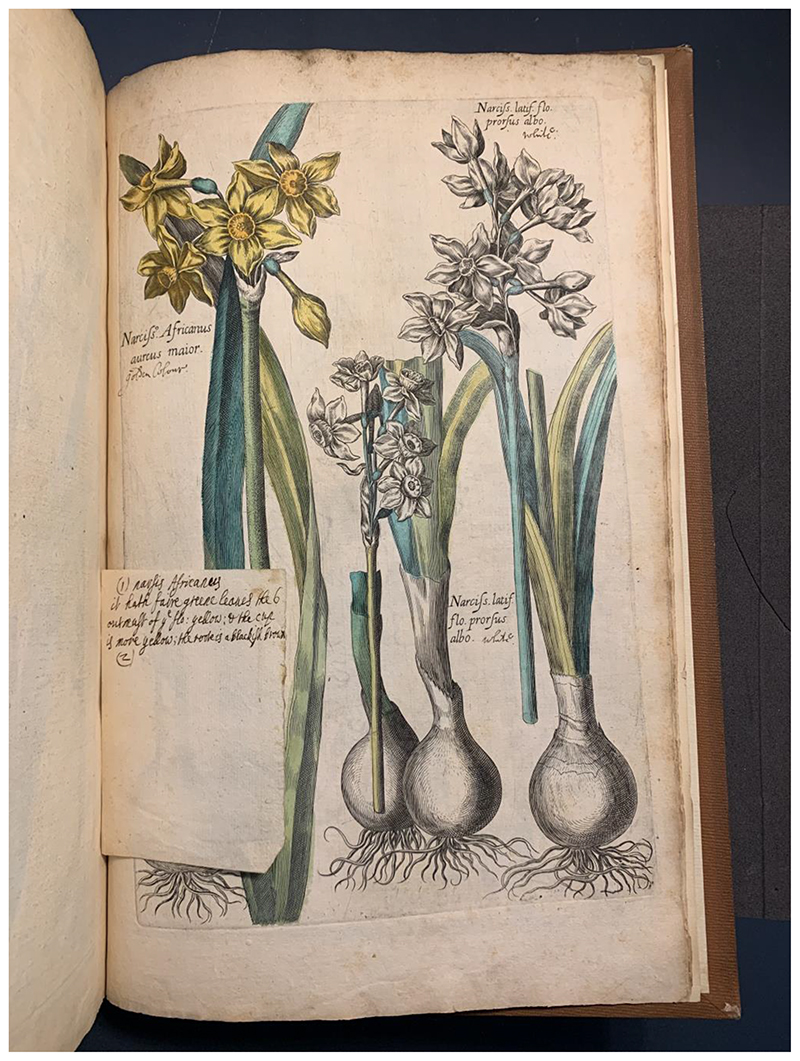
The page of *Narcissus Africanus* coloured by Margery, with her colour description slip and Herry’s annotations, in Johann Theodor de Bry, *Florilegium novum* (Frankfurt, 1612), British Library 442.g.11.

In Margery’s case, colouring flowers was not simply an easy pastime activity. To colour all these plants and flowers in a faithful-enough manner, Margery had to identify the species, understand the plants’ taxonomy, and make her way through various information about their colours. For example, the *Narcissus Africanus aureus maior*, or the great yellow Daffodil of Africa that Margery coloured in her copy of *Florilegium* was accompanied by a slip with colour descriptions in her hand (Fig. 2): “(1) Narsis Africanus. It hath faire greene leaves the 6 outmast of the flo: yellow; & the cup is more yellow; the roote is a blackish brown.” Although she was colouring a German florilegium, it is possible that this description actually came from Parkinson’s *Paradisi in Sole* (1629), listed as ‘Parkinson’s Garden’ in the 1664 book catalogue, as many other contemporary herbals, including Gerard’s, did not include this species. The key information in Margery’s slip was identical to Parkinson’s description of the same flower, as shown by the underlined phrases: “(their leaves were) a better greene colour, then many others that are grayish, among which appeareth a stalke, not rising to the height of the leaues, bearing at the toppe out of a skinnie hose many faire, goodly, and large flowers, … of a faire shining yellow colour, hauing large, round, and open cups or boules, yellower then the outer leaues; … the roote is great, and couered with a blackish browne coate or skinne”^xxiv^ The colours she used matched her own commonplaced information: she did colour the inner cup of the flower ‘more yellow’ than the outer pedals. In Margery’s process of colouring, bookish botanical knowledge based on Henry’s book collection was utilised, extracted and synthesised based on her artistic and natural-historical interests.

Margery’s labour, knowledge and creativity left their marks on both the botanical books signed with Henry Power’s name, and her own album of copied paintings and original drawings. She combined various printer sources to explore the figuration of one of her (presumably) favourite flowers: Tulips.^xxv^ British Library Add MS 5298 is a drawing album of 140 folios, including flowers, animals, tools and colour plates that were very possibly drawn by Margery and perhaps also by her drawing teacher, Henry Gyles. In this album, more than a hundred pages are drawings of tulips. The album is catalogued as “Tulips, Iris’s &c Birds &c done by Mrs. Power. Icone. in water colours, some in Oil. not bound” in Sloane 3972 Vol.VI, which indicates that these were probably originally separate pieces of drawings made by Margery and only bound later after being acquired by Sloane.^xxvi^

Judging from the sizes of the flower pictures and the black lead traces left within the ink contour, it is possible that Margery traced these pictures from printed books. Her sources include de Bry’s *Florilegium*, as pointed out by Kim Sloan, and the copy of Parkinson’s Paradisi in Sole, owned by the Powers, and potentially other flower books. Using black lead pencils to trace images has been a recorded technique since the second half of the seventeenth century. Odoardo Fialetti described the whole process in his *The whole art of drawing* (1660), including the use of Venice paper, oil, black lead pencils and charcoal. William Salmon in his *Polygraphice* (1672) also recorded the same technique using Venice paper, oil and black lead to trace images.^xxvii^ Margery certainly had all these necessary materials for tracing. On 5 July 1654, the day when Henry ‘was admitted Dr in Med in Cambridge’, Margery scribbled down a shopping list in Henry’s memorandum book asking him to buy her ‘one quire of royal paper, one quire of Venice quilt paper’ as well as other drawing equipment including cans and cups of tamarisk and serpentine tone colours, plus a magnifying glass.^xxviii^

**Fig.3:**
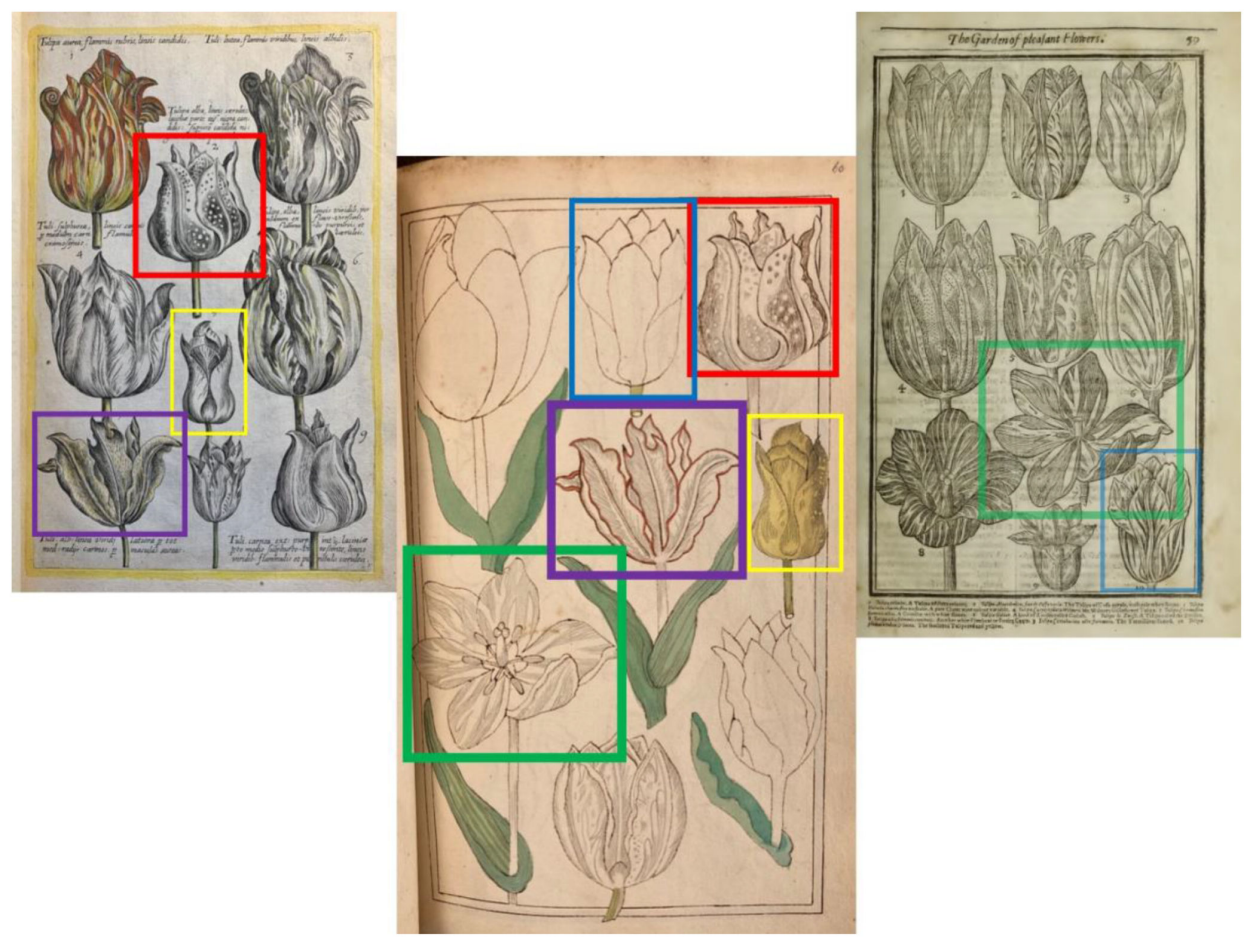
Margery’s tulip images after de Bry and Parkinson. Left: Johann Theodor de Bry, *Florilegium novum* (Frankfurt, 1612), British Library 442.g.11, unpaginated. Middle: Margery’s drawing combining flower figurations from both Parkinson and de Bry. British Library, Add MS 5298, f. 60. Right: John Parkinson, *Paradisi in Sole, Paradisus Terrestris: Or, a Choice Garden of All Sorts of Rarest Flowers* (London: R.N., 1656), 59.

Even though Margery might have been tracing, she curated the flower images from various sources according to her own design. Like the author de Bry himself, who copied images from other herbals onto his metal plates, Margery also combined tulips from different sources into one single image and tried to curate her own flower book, as shown in Fig.3. The tulip flower with a folded petal, for example, was clearly traced from page 59 of Parkinson’s *Paradisi*; but Margery elongated the stem, added a leaf, and arranged it in a different way.

Margery’s flower album contains several drawings of animals and flower patterns, many of which are almost identical to drawings from Ellen’s album, currently British Museum, 1975, U. 1589, with the signature “Ellen Power Booke” on the last page.^xxix^ These identical images may reflect the time they practiced drawing together. In Sloane’s catalogue, this album was recorded as “Several flowers fruits, Birds & Insects painted in water colours done by Mrs. Ellen Power in an Oblong. 4to,” which confirms Ellen’s authorship and ownership.^xxx^ Ellen’s album was initially bound together with a pattern book of beasts, fruits and flowers, which was made after Jacques Le Moyne’s *La Clef des Champs*.^xxxi^ Both Margery’s and Ellen’s albums included some images drawn after this pattern book, potentially also other pattern books.^xxxii^ They also referred to Adriaen Collaert’s *Avium vivæ icones* in their drawings, which provided models for parrots, peacocks, turkeys and many other animals.^xxxiii^

**Fig.4:**
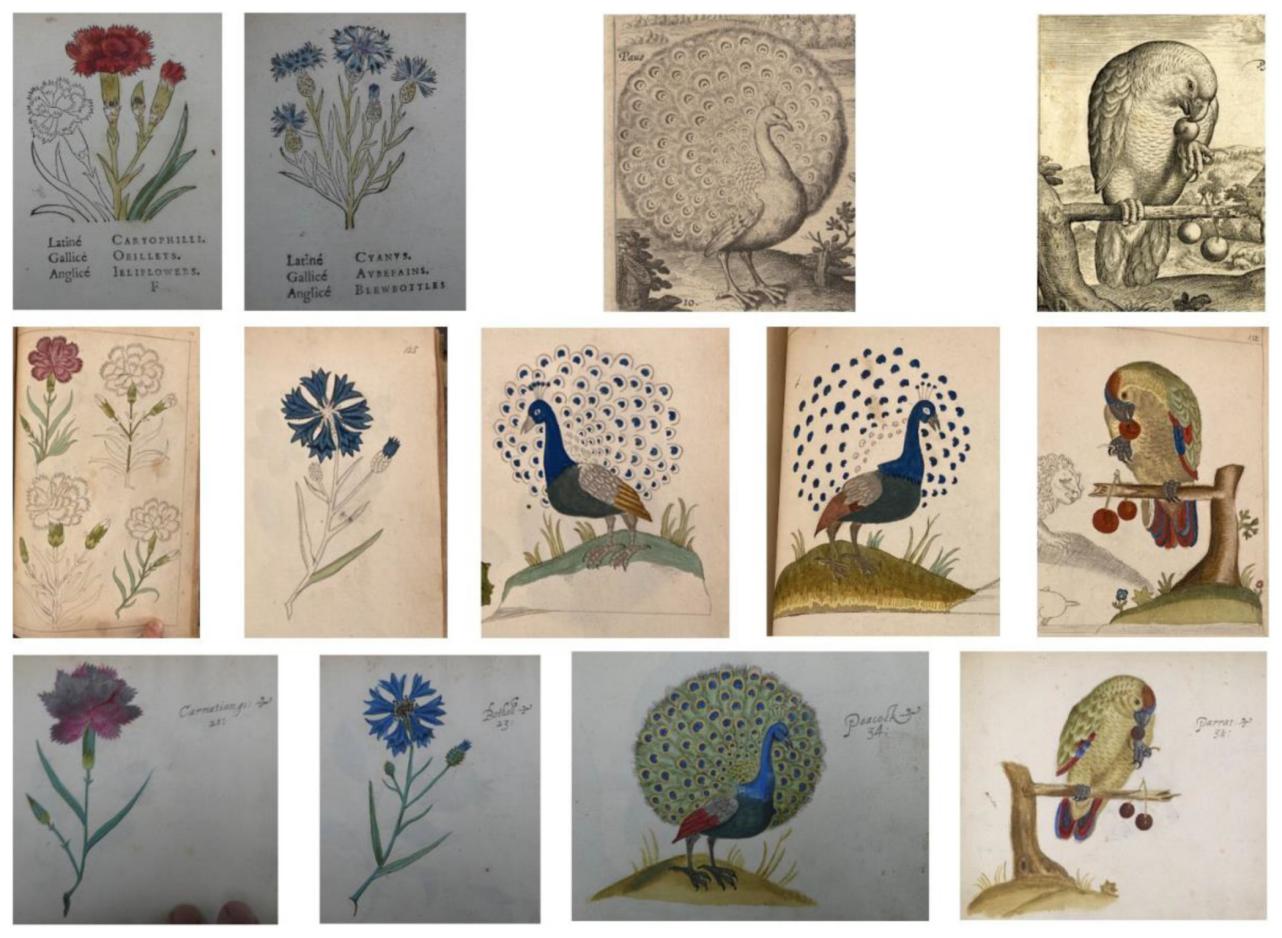
A comparison of the drawings of Margery and Ellen Power with their original sources. First row: British Museum, 1952, 0522, fol. 33, 25, after Jacques Le Moyne de Morgues, *La clef des champs pour trouuer plusieurs animaux, tant bestes qu*’*Oyseaux, auec plusieurs fleurs & fruitz* (Londre: Imprimé aux Blackefriers, T. Vautrollier pour Jaques le Moyne, dit de Morgues paintre, 1586); Adriaen Collaert, *Avium vivæ icones in æs incisæ et editæ ab A. Collardo* (Antwerp, 1610), two plates unpaginated. Second row: British Library Add MS 5298, fol.116, 125, 121, 131, 132. Third row: British Museum, 1975,U.1589, fol.21, 23, 34, 35.

**Fig.5:**
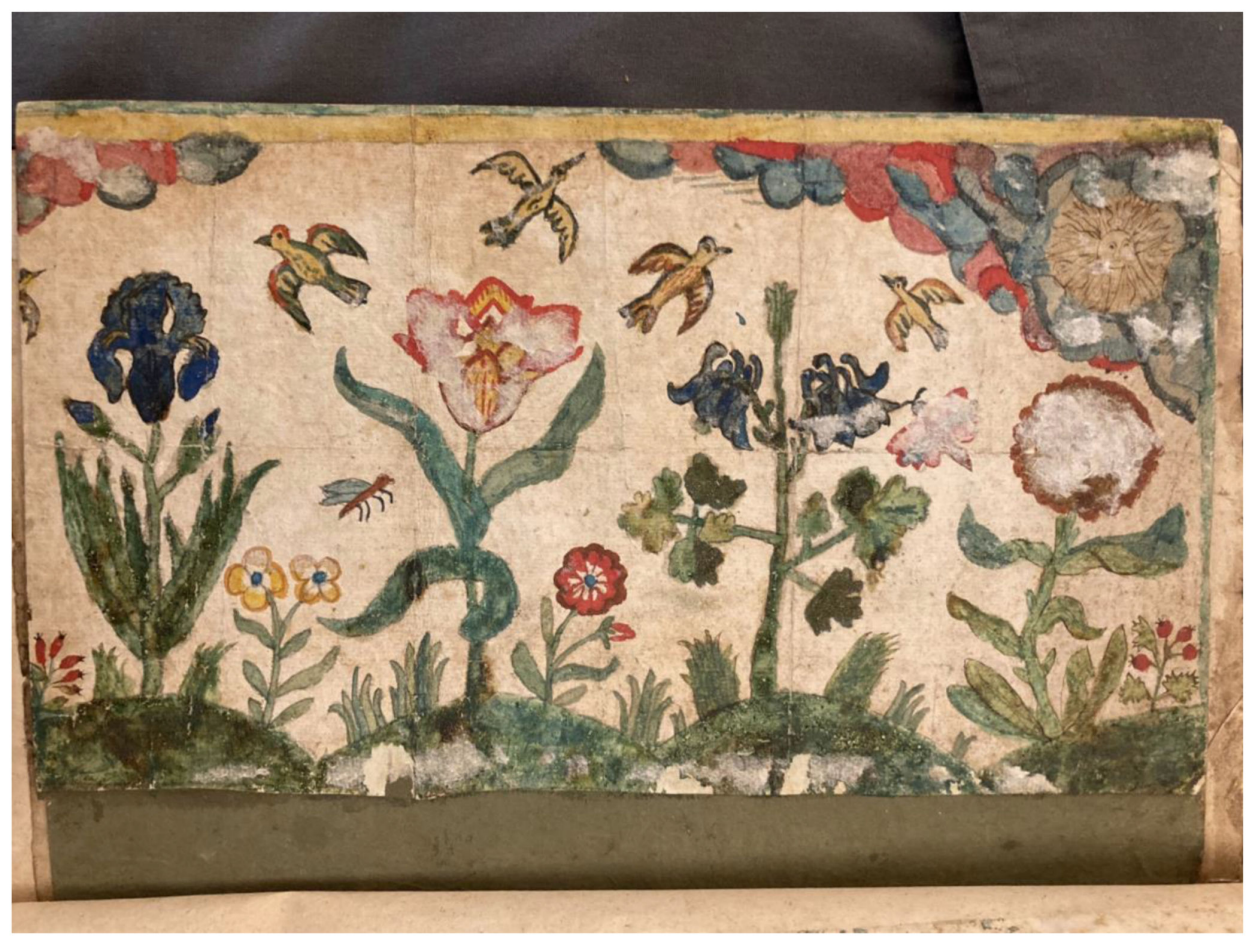
Margery’s painting. British Library, Add MS 5298, bound at the album’s end.

Based on various printed sources, the styles of Ellen’s and Margery’s drawings combined and switched between embroidery patterns and natural-historical drawings. Comparing their drawings with the potential sources (Fig.4) shows us how printed sources shaped their artistic styles. The two sisters presented great details and nuances when drawing after, possibly tracing Collaert’s parrot, and shifted to stylized, simple forms when creating flower patterns. When creating a piece of art themselves, all these styles could be utilized creatively in combination, as shown by the drawing bound at the end of Margery’s album (Fig.5). It is a watercolour on hard paperboard, depicting a delightful scene of flowers, birds and insects living together under the bright sunshine. Compared with other surviving seventeenth-century English embroideries depicting natural scenes, the style, composition, and glue-stuck traces on paper indicate that this image by Margery may have been used as a template underneath an embroidered picture. ^xxxiv^ Margery created figures of iris, tulips, marigolds, and other plants based on what she learned from de Bry’s, Parkinson’s, and Le Moyne’s images. The form of the Columbine, for example, was very likely after fol. 33 of Le Moyne’s pattern book, while the realistically depicted Iris flower was possibly the result of the numerous drawings of Iris in her album book. Knowledge from flower books and embroidery manuals both became Margery’s source of artisanal creation.

Stories surrounding the women’s books in the Power household reveal more historical nuances beyond individual book ownership. Unlike many early modern husbands, Henry seemed to acknowledge his wife’s ownership of her book. Apart from cataloguing the book as his wife’s, he also signed Margery’s name on the book several times on her behalf: the giant size “Margery Power 1657” on the book lining, the small signatures of the couple both on the hand-coloured, gold-leaf-decorated frontispiece, and the brackets-framed “Margery Power Her Booke” at the end of the preface of this *Florilegium*, were all in Henry Power’s hand. Moreover, Henry also took care of Margery’s book. In a bill dated 10 October 1656, Henry noted that he “paid for my booke vamping” and the vamping of “my wife book” each five dimes; the latter could very possibly be this *Florilegium* scrapbook or other books owned by Margery that we do not yet know of.^xxxv^ This reveals a complex dynamic in the Power household regarding the ownership of books. On the one hand, Henry acknowledged Margery’s ownership of this book, and went out there to help Margery mend her book; on the other, Henry still signed his own name on Margery’s book, and listed the book in “A Catalogue of all my Bookes” along with his other 507 books. After Henry’s death, Margery still catalogued her husband’s books as “A Catalogue of Dr Powers Bookes,” though the entry “My wifes Booke,” or the *Florilegium* book was taken out of this version of the catalogue.

In Margery’s case, even though she owned few books as an individual, her intellectual pursuits in reading were nonetheless very present in her album of botanical illustrations, her hand-coloured herbals, and her botanical notes on the slips hidden inside the books. While it has been pointed out that early modern women’s books were often listed as a part of household property, the libraries of learned men such as Henry Power should not be merely seen as their own either. It was not a private library, but a household library in its nature, and a place for collaborative reading, learning and experiments. The library of the Power household shows another “ownership” of books, one that was not determined by the number of books owned by an individual, but by the intellectual endeavour shared among female and male household members.

## Everything Has Many Lives

In Sloane 1343, a handy little notebook with extensive reading notes on the botanical tradition from the time of Theophrastus to Parkinson’s *Theatrum Botanicum*, Henry Power scribbled down a poetic reflection on the value and use of plants as a preface.^xxxvi^ According to this preface, plants and their products constituted a lively and dynamic economy:

For by the medium of Plants god has bestowed all food clothing & medicine upon man. To this ofspring wee also ow (for the most part) our Houses, shipping & infinite other things though some of them Proteus-like have run through divers shapes, as this paper whereon I write, that first from seed became flax, then after much exation thread, then cloth, where it was cutt & mangle’d to serve the Humours & fashions of Men.^xxxvii^

Henry’s acknowledgements that plants entered a cycle of being used and reused from the very starting moment of their life shows that Henry, like his contemporaries Issac Newton and John Beale, really was a man living in the seventeenth-century ‘cyclical cosmos’, as analysed by Simon Werrett.^xxxviii^ Henry was fully aware that papers and books, those media of his plant knowledge, were originally plants as well. From seeds and flowers to medicine made from them, from paper to books and notebooks, this section will focus on the economic life of plants in the Power household, putting materials, books and manuscripts under the same lens of recycling. It will show how recycling functioned as a form of collaboration, and how knowledge was produced and transmitted through the recycle of plants in the Power household.

Paper was always recyclable in the household of Henry and Margery Power. As we examined before, various volumes of de Bry’s *Florilegium* were torn up, bound into a new volume for Margery to colour. So was the paper Margery used for drawing. The Venice paper that Margery used for tracing was also a popular tool for observation among contemporary natural philosophers: Walter Charleton affirmed that a granule of dust, when observed “on a sheet of Venice Paper,” appeared to be “conflated of ten hundred thousand millions of insensible Particles.” Similarly, Robert Hooke also advised using “a piece of very fine Venice paper” as the background for observing the fracture of sunbeam in the water.^xxxix^ For microscopists of her time, Venice paper was a very good backing paper for its smoothness and transparency. Henry Power also used paper in similar ways. He used “a white sheet of paper” to hold the falling spark from the striking flint and steel, in order to observe the “little dark spot or moat, no bigger then a pins point, which through our *Microscope* did appear to be a perfectly round ball polished like Steel or Glass”; when cutting a frozen eye to observe the interior structure, he also used “an oyled paper” to hold the object.^xl^ We do not know for sure, though, whether Henry borrowed his wife’s ‘quire of Royal paper’ and ‘quire of Venice guilt paper’, which seemed to be high-quality judging from the paper used in her drawing album; it is not surprising if he did.

So were their notebooks recycled. The memorandum books that Henry and Margery used were already written in a thrifty manner: written from the beginning to the middle-end in one year, and two years later, flipped upside-down and used from the end back to the beginning. As we have shown, Margery kept using the same notebooks after Henry’s death. In the Power household, recycling was always inseparable from collaborating. In the same notebook that Henry wrote his canon of botanists, notes on *Theatrum Botanicum* and opinions on plant-made paper, two more people also wrote in it. Following Henry’s botanical commonplaces without leaving many blank pages in-between, someone started copying down several pages of recipes from Gerard’s herbal in a neat hand. Sloan has suggested that these recipes could be written by Henry’s wife, yet this hand is very different from both Margery’s and Henry’s tilting, elongated Italic hands.^xli^ These recipes were written within the grids that Henry drew in his notebook, and intersecting with Henry’s: after this compiler had been commonplacing recipes for around ten pages, Henry started to write again, noting down a remedy for gonorrhoea. It is possible that these recipes were from other family members or commissioned by Henry or Margery to their servants to copy down. We do not completely exclude the possibility, though, that this could be Henry’s earlier, more rigid hand from his student time.

Either way, these recipe notes from Gerard reflects the Power couple’s common interest in flowers. An object for artistic and natural-historical interests, the flower received particular attention in the Power household. Suppose that a lively flowering lily by some coincidence fell into the hands of the Power couple, it could face several destinies: It could be dissected on the table, have its long style removed, and pendants tipped off to be “pleasantly” observed through one of Henry’s microscopes and reported in the *Experimental Philosophy*.^xlii^ Or, its flower could be “put into a glasse, & set into a hill of Ants”, closed and stopped for a month there until it turned into a liquid to be applied outwardly for gout, following a recipe in Henry’s *Theatrum Botanicum* notebook commonplaced from Gerard’s herbal.^xliii^ If lucky, it could also survive as a whole plant, be put into a vase and used as Margery’s model for drawing and colouring, as she might do with flowers in their garden. Other plants were similarly used by both members of the family. While Margery was colouring and drawing poppy flowers and strawberries in her album, Henry was putting their leaves and seeds under the microscope. ^xliv^ In the botanical world of this couple, flowers were always used in collaboration and for diverse purposes.

It is certain that Margery and Henry had a garden in their house at least since their move into New Hall in 1656. As Jennifer Stead has documented on the basis of a reading of Henry’s memorandum books, the Power’s spent most of their years at New Hall renovating the gentry house, its garden, and its outer walls.^xlv^ In October 1661, Henry paid a waller named Aspen to renovate their greenhouse, and in January 1662/1663, he paid a hedger named Hallam to plant 170 trees on their land. Hallam was also hired as a gardener to take care of the root garden, the knot garden, and the garden patch under the window of their renovated kitchen.^xlvi^ Unfortunately, Henry does not mention which plants they nurtured in the greenhouse or the exact species growing in the several garden patches. It is likely that he grew medicinal plants himself, though, for he notes down that he had a joiner make shelves and nests of boxes for his collection of *materia medica*, including a number of long drawers for roots.^xlvii^ It is, then, very possible that the seeds of corn poppies, maidenhair, strawberries and moonwort that Henry observed through his microscope were later planted into the gardens maintained by Margery and their gardener Hallam.

In the Power household, flowers, leaves, fruits, and spices used in the kitchen, as well as other commonly used plant products were both objects for consumption and objects for research. As scholars such as Elaine Leong, Wendy Wall and Simon Werrett have pointed out, culinary art and knowledge of food played an important part in early modern experimental science and medicine.^xlviii^ Henry seems to have been very interested in culinary art: Sloane 1319, a quarto notebook of more than thirty folios, is the result of his encyclopaedic effort in culinary knowledge from plants’ medicinal virtues to ale making. Beginning with common bread-making materials such as wheat, rye, and barley, to spices such as rosemary, parsley, and coriander, to the flesh of beef, goats and hares, Henry listed specifically the temperament and degree of all these common foods, which diseases they were good for, and how to process them medicinally. Many of the flowers and fruits Henry studied in this book, such as marigold, rose, violets and turnips, pears, cherries, strawberries and so on, were also painted in Margery’s drawing album. While Margery’s drawings were from printed sources rather than directly from nature, her selection of species reflects that fruits and flowers commonly available in the household also spurred her interest. Reading and colouring those plant illustrations, Margery learned about both the curious exotic tulips and England’s most common plants and products, the latter of which became materials for both Margery’s drawing and Henry’s experimental research. The poppy seeds, lilies and strawberries we find in Henry’s microscopical observations show how his research was conditioned by his familiarity with everyday objects and their characteristics in the household context.^xlix^

Like plants in the garden, books and paper also carried multiple functions in early modern households. Historians of graffiti have shown how a book in an early-modern family could be used simultaneously for pen trials, calligraphic teachings and exercises. ^l^ In Margery’s case, the books were also her laboratory of artistic experiments. Instead of seeing books as mere books, she and her husband also saw them as separable pages and tearable paper. Her compiled copy of the *Florilegium* made of images from various editions can be seen as an extended form of extra-illustration, a practice of collecting visual materials to insert into and enhance existing printed books flourishing mostly since the eighteenth century. A unique way of reusing paper materials, extra-illustration was usually considered as a pastime activity for rich gentlemen, which required extra investments in collecting images and sometimes rebinding books.^li^ While Werrett has pointed out the importance of thriftiness in household science, for the affluent Power family, their “recycling” was more likely for epistemic purposes than economic purposes.^lii^ It was not a thrift, but a luxury, that Margery and Henry were able to recycle a 14s flower book.

One used something for a purpose, the other used it again for another — this kind of recycling was also an important part of the collaboration between Margery and Henry. This can be seen from the annotations and commonplaces from the herbals in their household library. Both of the two books Margery coloured, *Florilegium* and *Icones Stirpium*, were annotated by both Margery and Henry. Henry’s annotations almost covered every entry from the beginning to the end, while Margery’s slips and handwriting appeared occasionally in some pages. It is possible that they read these books together, or they could also have been taking turns, one working on it after another.

Like many of his contemporaries, Henry’s comprehensive annotations showed that plant names and references were his main concern.^liii^ While annotating *Icones Stirpium*, Henry translated Latin and foreign-language plant names into English and cross-referenced to other English and continental herbals, occasionally adding some findings from his own observations. On page 309, he noted that the two English names of gentian minor were from “Ger. Chap.101.” On page 200, on “Irio Sive Erisimum Dioscoridis,” he noted: “Bank Cresses. So called first by Dr. Turner. Vid. Gerard. Chap.15. pag.197.” As Gerard did not refer to Turner in his entry, it seems that Henry was examining multiple herbals while checking the images of one plant, including William Turner, Pietro Andrea Mattioli and other herbalists.^liv^ This taxonomical interest corresponds with his way of reading Parkinson’s *Theatrum Botanicum*. In the botanical notebook Sloane 1343, Henry noted down a detailed index of every “tribe,” or category of Parkinson’s herbal and plants listed in those categories: in the tribe of Cathartic or purging plants, for example, he listed the name of Aloe, Indian Juice, Rhubarb, Coolquintida etc., all according to Parkinson’s original sequence.^lv^

**Fig.6:**
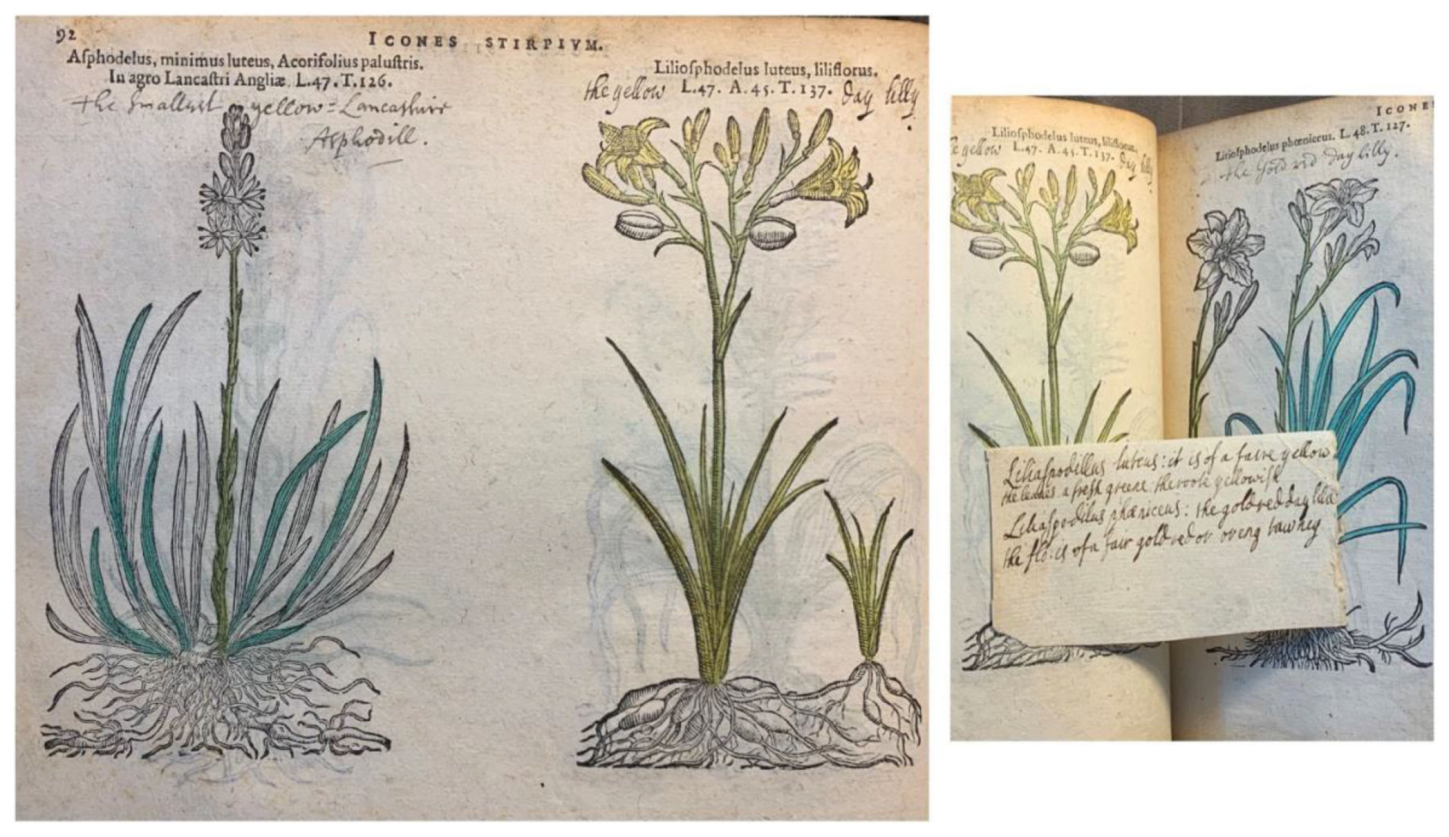
Matthias de L’Obel, *Icones stirpium, seu Plantarum tam exoticarum quam indigenarum, in gratiam rei herbariæ studiosorum in duas partes digestæ cum septem linguarum indicibus, etc*. (Antverpiæ: Ex officina Plantiniana, 1591), 92-3. With a slip attached in between. British Library, 443.a.5.

While Henry read Parkinson’s *Theatrum Botanicum* most carefully, Margery frequently referred to the descriptions from Parkinson’s *Garden* for colouring, as analysed in the first section. Like Henry’s annotations, her numerous slips were also written in a consistent manner, containing the names, variants, and colours of each part of the plant — flowers, leaves, roots, and so on. Sometimes Henry might have been working with her, as there are also slips and annotations on plants’ colours in Henry’s hand; but it is not clear whether Margery coloured them first, or Henry annotated them first. Usually Henry’s annotations on colour contain only one or two words, ‘yellow’ or ‘white’, while Margery’s slips contain more information and nuances, as much as in her colourings. On page 92, (Fig.6) in *Icones Stirpium*, we can find a moment when both of them performed the botanical task of translating plant names: Henry’s hand ‘Englished’ the two flowers “the smallest yellow Lancashire As phodill” and “the gold red day Lilly,” while Margery’s hand ‘Englished’ the flower in the middle “the yellow day lily.” A slip Margery wrote was also inserted here, about the yellow lily on this page and the gold red day lily on the facing page: “Liliaspodillus luteus: it is of a faire yellow the leaves a fresh Greene: the roots yellowish; Liliaspodilus phoeniceus: the gold red day lilie the flo: is of a fair gold red or orang tawney.”^lvi^ Her husband noted down the English name of the golden lily, but Margery used the Latin name in her slip, and coloured the yellow lily yellow and its stem fresh green accordingly. It is possible that their annotations resulted from different but continuous rounds of research, one recycling the information produced by the other. Despite their different interests, both of them entered the common seventeenth-century botanical practice of extracting and reorganising the names and characteristics of plants, with taxonomical awareness of the differentiation of species. Through rounds of engagements with texts, images and plants, Margery and Henry established a cycle of collaboration and knowledge production in this household.

## Creating colour

Towards the end of the first book of *Experimental Philosophy*, Henry Power lamented about the difficulties he faced when trying to establish the colours of his microscopical specimens: “most, if not all, Bodies in their minute particles (through which the Sun’s Rays have more freedome to penetrate) seem to lose their Colours, and grow diaphanous, as you may observe in the *Microscope*.” ^lvii^ Similarly, Henry argued that the reason that insects and fish traditionally had been considered to be bloodless was because their blood vessels were so fine that they only contained a tiny bit of coloured material and not enough to give off a proper red colour.^lviii^ Seeing colours through microscopes was indeed difficult.^lix^

And yet, in the individual microscopical observations of the *Experimental Philosophy*, we nevertheless find an abundance of colour: The powder of the white lily “looks of a pure pale yellow, and like so many pieces of polished Amber,” the seeds of strawberries “look not unlike the Strawberry; some reddish, yellowish, and green colours, as the Strawberries themselves are,” the livers of lampreys are “a pure grass-green colour, which (…) presently turned into a faint Olive-colour,” while the eyes of the little white short-legged spider had “a pale yellow circle, which encompasseth a violet-blew Pupill, most clear and most admirable.” ^lx^ Although he complained that the task was difficult because of the tendency to opaqueness, in his descriptions Power revealed a heightened sensibility towards colour and an advanced vocabulary with which to describe them.

Before the eighteenth-century emergence of taxonomical systems based on morphology, descriptions of the colours of *naturalia* was an important part of any identification of specimens, and especially plant specimens. ^lxi^ As argued by Valentina Pugliano, colour sensibility – that is the ability to differentiate between the many tones and hues of colours and to describe them using a somewhat stable nomenclature – was crucial for the naturalist in general, and for the microscopist in particular. We should remember that the microscopist was seeing coloured objects that no one had described before, so a chromatic vocabulary of precision and accuracy was all the more important. But unlike shapes and sizes, which were easily described using the language of geometry, colour was a more fleeting quality, and, as the artist and natural philosopher Richard Waller pointed out in 1686, a “Standard of Colours” was yet a “thing wanting in Philosophy.”^lxii^ Thus, naturalists had to rely on the experience of those who mastered colour, such as artisans, painters, chymists and craftsmen.^lxiii^ In this section, we show that attempts to develop a chromatic standard were undertaken within the Power household through experiments with colour production.

**Fig.7:**
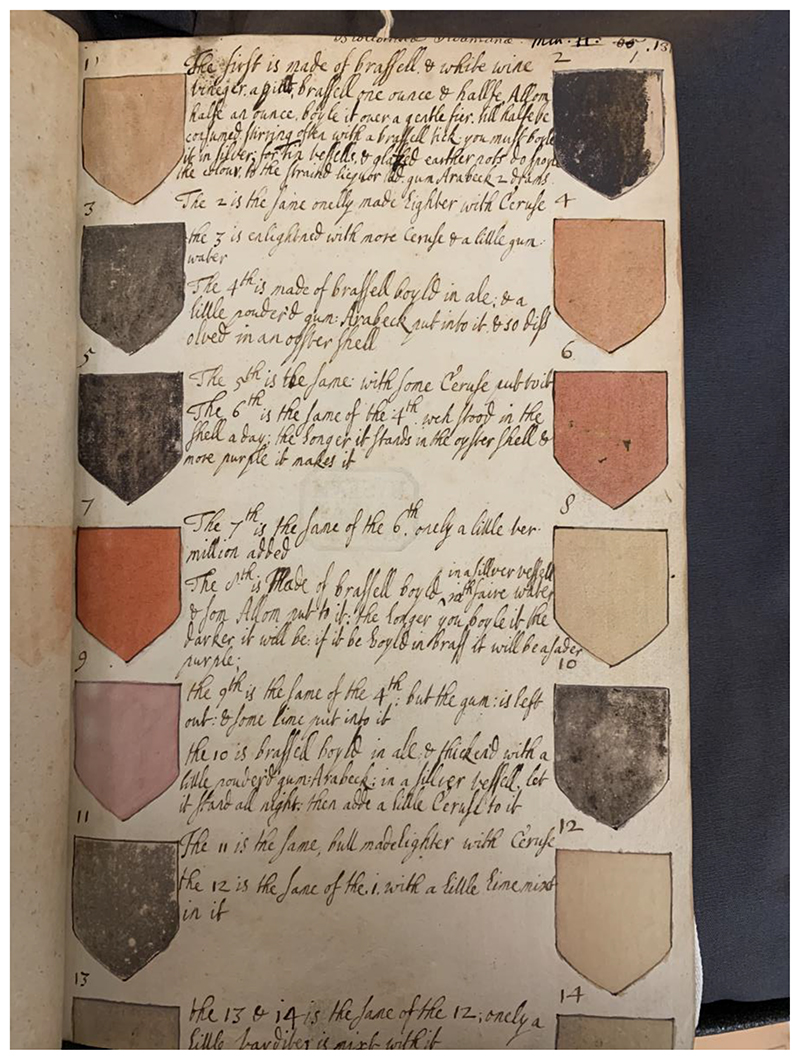
The colour shields in Margery’s drawing album. British Library, Add MS 5298, f.1.

Margery was well aware that colours could never be categorised simply by single terms like green or red; she knew that there is a variety of tones in every colour. In her notes on the colours of plants that she extracted from her reading of herbals, she mentioned, for instance, a “blackish blew,” “a perfect blue colour, tending to purple,” “a fair gold red or orange tawney,” and a “whitish greene colour.”^lxiv^ And looking carefully at Margery’s hand colouring, we can see that while she used the same two kinds of greens for most of the flowers, she also mixed these greens to produce either a lighter or a darker green. From their memorandum books, we know that Margery had Henry buy her serpentine tone, a kind of green, from the market, but she would have adjusted the tint of this colour to make it lighter or darker, more yellow or more blue depending on her needs.^lxv^ Margery also made her own colours from scratch using a variety of ingredients, tools and manufacturing processes.^lxvi^ On the first two pages of her watercolour album, Margery wrote up her recipes for making coloured ink (Fig.7). Embellished with a number of coloured shields on each side of the margin, Margery detailed how to produce these colours using a variety of highly expensive ingredients. Unfortunately, the pages seem to have been cut up in order to fit the size of the album, which means that 12 of the coloured shields and recipes are missing, but we are still able to get interesting insights into Margery’s practice of producing colours.

To make her colours, Margery used white wine vinegar, alum, ceruse, gum Arabic, ale, vermillion, lime, sugar candy and logwood water. In order to turn these materials into coloured paints, she either dissolved them in an oyster shell or boiled them in a silver vessel. While Margery did not specify where she had learned to make colours through these methods, her materials, instruments and procedures resemble those circulating in manual books and books of secrets at the time.^lxvii^ The processes of colouring were described in books such as Henry Peacham’s *The Gentleman Exercise* (1612) and John Bate’s *The Mysteryes of Nature, and Art* (1634), just as Margery might have learned about colour tinting in the anonymous cosmetics treatise *Artificiall Embellishments*, which included recipes to produce make-up using the same materials as those of Margery.^lxviii^

Although some of the coloured shields are now faded, we can see that Margery successfully produced some beiges, two slightly different reds, two tones of pink, a blue colour and a purple colour. Reading through the recipes, it is apparent that many of the colours are variations of each other. For instance, Margery wrote that “The 2 is the same onelly made lighter with Ceruse,” “The 7^th^ is the same as the 6^th^ onely a little vermillion added”. She also described that the 6^th^ colour, a beige-cum-red, was the same as the fourth, which had stood in the oyster shell a day, which had caused it to turn darker: “the longer it stands in the oyster shell & more purple it makes it.”^lxix^ This method of letting the solutions stand for a shorter or longer period in order to reach the desired hue was also generally reported on in the art manual literature, as was the process of adding small quantities of another substance such as ceruse or vermillion.^lxx^ In total, Margery listed recipes for the making of 31 colours, but many of these were slightly different nuances of the same colours, such as beige or red. These careful experiments tell us about the familiarity with which Margery manipulated colour.

As such, the colour catalogue presented at the beginning of the watercolour album served three purposes. First, it allowed Margery to compare colours easily by presenting them in an organised way on two pieces of paper. Second, by giving the recipes for making the colours, it would have allowed her to recreate them at a later stage in order to ensure some consistency in her homemade colours. But, third, just as with Margery’s medical recipes, the purpose of these colour recipes was not only practical. Rather, they show Margery’s interest into the ways in which it was possible to change colour through the use of chemical substances and procedures and as such they constitute an enquiry into the nature of colour itself not at all that different from the debates on colour taking place among her male peers.

Margery shared this interest into the nature of colour with her husband Henry, who produced colours using procedures, techniques and ingredients with a high degree of resemblance to Margery’s. In the fourth deduction at the end of his book on microscopy, Henry discussed the nature of colours and concluded that colours are nothing but the effect of the specific motion of the ‘solary atoms’ following the arguments made by René Descartes in his *Météores* and *Principia*. In the *Météores*, Descartes had experimented with glassware in order to create ‘artificial’ rainbows thereby establishing his spin theory of colour.^lxxi^ Henry agreed with this theory and argued that “Colour is nothing else but the modification of Light, which by the alteration of its motion is dyed into colours.”^lxxii^ In order to expand on this theory, Henry included a number of experiments in which he altered the colours of liquids by adding different chemical and vegetable solutions to it. For instance, Henry added drops of the oil of tartar to an infusion of violets to create a green liquid, which he then changed into purple by adding some oil of vitriol.^lxxiii^ He also changed a red infusion of dried roses into green by adding spirit of harts-horn, and made amber colour by adding “a little sharp vinegar” to an infusion of Brazil wood.^lxxiv^

Interestingly, like Margery’s catalogue, these experiments are all given as recipes as they give instructions to ‘you’, the reader, on how to create the different tinctures. And like Margery, Henry is also interested in how to change the tone of colours: If a few drops of the oil of tartar is added to the “reddish Infusion” of Brazil wood, “it will turn it to a pure purplish red, like excellent Claret.” ^lxxv^ Henry also noticed that when specific chemical substances were added to the coloured liquids, they turned transparent only to become colourful again when another substance was added. This experiment strongly resembles the chemical experiment referred to as the ‘reduction to the pristine state’ that Robert Boyle published in his 1666 *The Origine of the Formes and Qualities*.^lxxvi^ Here, too, Boyle argued that even though the appearance of a chemical solution, such as its colour, could be altered by adding different substances, it nevertheless remained the same.^lxxvii^

Henry’s own experience with colours and what he learned from Margery not only allowed him to identify and describe the colours that he observed through his microscopes; they also carried significant epistemic importance. In his discussion of chicken generation in *Experimental Philosophy*, Henry revisited the observations made by William Harvey on the first appearance of the heart and blood within the chicken egg.^lxxviii^ Henry concluded that it was Harvey’s lack of attention to colour changes that had made him fail to recognise that the heart, including the auricles and ventricles, was actually present long before he had claimed, although in a colourless, transparent state. What Harvey had taken to be the gradual appearance of the chicken, Henry recognised merely as a change in colour from white to vivid red rendering the already-present heart visible. Thus, a “pretty and beneficial Observation of the *Microscope*” showed him that when the chicken heart did appear as a “red pulsing Particle,” it was already fully formed.^lxxix^ The awareness of the changing colours thus allowed Henry to develop a new theory of generation that emphasised the preformation of bodily organs.

The sensibility towards delicate colour changes, the standardised language of colour description and the chymical procedures of making and testing colours: it would be hard to distinguish Margery’s and Henry’s experiments with colours, were it not the case that one of them appeared in a celebrated publication of microscopy, the other in a forgotten manuscript. Even their ingredients were very similar: when Margery used an infusion of logwood with vinegar, Henry used the almost-identical Brazil wood. They both had an interest in creating a variety of colours and different tones of colours, and both of their practices speak to a deep knowledge of the malleability of colours and an ability to recognise individual colour tones. ‘One drop’, two drops, they both consciously controlled the amount of substances they were adding, observed the effect, and carefully recorded the process. For Margery, these colour trials were not only a practical procedure, but also an epistemic experiment aiming to understand the nature of colours, and learn how to control colour changes.

## Conclusion

Throughout this paper, we have traced how botanical information travelled in the Power household from herbals to paper slips and recipe notebooks, to Margery’s drawing album, and Henry’s observations in the *Experimental Philosophy*. Books, together with the surviving library catalogues of the Power household, served as an important clue and medium of the story. Tracing their collaborative use of books, we argued that the books of the Power household were a household library shared between Margery and Henry, serving practices that oscillated between being practical, artistic and scientific. The textual botanical knowledge went hand in hand with Margery and Henry’s practical knowledge, which enabled them to explore common household plants for various epistemic purposes, from drawing and observing to medicine-making. By controlling colours, developing a chromatic vocabulary and standardising ways of plant colouring, Margery created a sensibility of colours that was significant for microscopical observations.

Unlike Henry’s *Experimental Philosophy*, Margery’s work on the colours of plants was never published or otherwise disseminated until it ended up in the British Library together with the rest of the Power marginalia. Moreover, unlike Henry, whose introduction to the world of book printing and public letter-writing had been facilitated by the university education exclusive to men, Margery did not consider herself an author. However, her artistic collaboration with Ellen, as well as all the botanical activities that she undertook on her own and with Henry, shows us that she was deeply engaged in an intellectual, experimental approach to nature and plants. This should remind us that it was not only when the work of the women of the household ended up being published – as it happened in the homes of the Listers, the Breynes and Réaumur – that these women made significant epistemic contributions.

In one way, though, can Margery’s work be said to have left the household. When Henry published his microscopical observations as the first book of *Experimental Philosophy*, no stable model for reporting such observations was established. Whereas Henry looked towards the genre of the books of Sir Thomas Browne, William Harvey and Thomas Moffett for inspiration on how to compile his findings, his way of seeing, as we have argued, was developed in tandem with Margery. This particular way of seeing involved a heightened sensibility towards colour, colour changes, the inherent opaqueness of small things, and a general fondness for local flowers that could be grown within or near the household. In the years following the publication of *Experimental Philosophy*, this approach to studying plant minutiae was continued by Robert Hooke and Nehemiah Grew, who both read and engaged with Henry’s work. Thus, in *Micrographia* Hooke makes observations of the powder of lillies and poppy seeds, while also comparing the protrusions on the face of a blue fly with “the Pendants in Lillies.” ^lxxx^ As he was getting used to working with microscopes, Grew, furthermore, took extensive notes on Henry’s observations in his commonplace book, and incorporated this way of describing into his published *Anatomy of Plants*. Here, he described how the “minute Particles” “of a *Tulip* or a *Lilly*, being larger, is more distinctly observable,” and that these powders were yellow or white, but that “each *Globulet*, through a Glass, [is] transparent as Crystal.”^lxxxi^ In this way, Margery and Henry’s attention towards the colours of plants travelled beyond their household.

## Footnotes

i For biographical details about Henry Power, see C. Webster, “Henry Power’s Experimental Philosophy,” *Ambix* 14, no. 3 (October 1967): 150–78; John Hughes, *Henry Power of Halifax: A Seventeenth-Century Physician and Scientist* (Oxford: Rimes House, 2010). Throughout this article, we refer to Henry Power as Henry, Margery Power as Margery, and Ellen Power as Ellen. This is merely to avoid the confusion of too many Power’s.

ii British Library, Sloane MS 1357, f.48 and f.58.

iii ‘After dinner up to my chamber and made an end of Dr. Power’s booke of the Microscope, very fine and to my content, and then my wife and I with great pleasure, but with great difficulty before we could come to find the manner of seeing any thing by my microscope’. The Diary of Samuel Pepys, Sunday 14 August 1664. Accessed 29 September, 2022.

iv Kim Sloan, “Sir Hans Sloane’s Pictures: The Science of Connoisseurship or the Art of Collecting?” *Huntington Library Quarterly* 78, no. 2 (2015): 381–415.

v Deborah E. Harkness, “Managing an Experimental Household: The Dees of Mortlake and the Practice of Natural Philosophy,” *Isis* 88, no. 2 (1997): 247–62.

vi Alix Cooper, “Picturing Nature: Gender and the Politics of Natural-Historical Description in EighteenthCentury Gdańsk/Danzig,” *Journal for Eighteenth-Century Studies* 36, no. 4 (2013): 519–29; “Natural History as a Family Enterprise: Kinship and Inheritance in Eighteenth-Century Science,” *Berichte zur Wissenschaftsgeschichte* 44, no. 2 (2021): 211 –27; Mary Terrall, *Catching Nature in the Act: Réaumur and the Practice of Natural History in the Eighteenth Century* (University of Chicago Press, 2014); Anna Marie Eleanor Roos, *Martin Lister and His Remarkable Daughters: The Art of Science in the Seventeenth Century* (Bodleian Library, University of Oxford, 2019). For the notion of the “four-eyed sight” of nature, Lorraine Daston and Peter Galison, *Objectivity* (New York: Zone Books, 2010), 84–98.

vii Florike Egmond and Sachiko Kusukawa, “Circulation of Images and Graphic Practices in Renaissance Natural History: The Example of Conrad Gessner,” *Gesnerus* 73, no. 1 (November 6, 2016): 29–72; Sachiko Kusukawa, “Picturing Knowledge in the Early Royal Society: The Examples of Richard Waller and Henry Hunt,” *Notes and Records of the Royal Society* 65, no. 3 (September 20, 2011): 273–94; Sietske Fransen, Katherine M. Reinhart, and Sachiko Kusukawa, “Copying Images in the Archives of the Early Royal Society,” *Word & Image* 35, no. 3 (July 3, 2019): 256–76..

viii Janice Neri, *The Insect and the Image: Visualizing Nature in Early Modern Europe, 1500-1700* (Minneapolis: University of Minnesota Press, 2011), chap. 5; Mary Learner, “Embroidering the New Science: Seventeenth-Century Florilegia and Botanical Study,” *Nuncius* 35, no. 3 (2020): 685–717.

ix James Clifton, “‘I Am Come into My Garden’: The Canticle of Canticles and the Florilegium of Philips Galle and Adriaen Collaert,” in *The Art of Biblical Interpretation: Visual Portrayals of Scriptural Narratives*, ed. Heidi J. Hornik, Ian Boxall, and Bobbi Dykema (Atlanta: SBL Press, 2021), 44.

x Sachiko Kusukawa, “The Early Royal Society and Visual Culture,” *Perspectives on Science* 27, no. 3 (June 21, 2019): 351; Meghan C. Doherty, “Discovering the ‘True Form:’ Hooke’s Micrographia and the Visual Vocabulary of Engraved Portraits,” *Notes and Records of the Royal Society* 66, no. 3 (2012): 211–34; Janice Neri, “Between Observation and Image: Representations of Insects in Robert Hooke’s *Micrographia*,” in *The Art of Natural History: Illustrated Treatises and Botanical Paintings, 1400-1850*, ed. Therese O’Malley and Amy R. W. Meyers (New Haven: Yale University Press, 2008), 83–107.

xi Brian W. Ogilvie, “Image and Text in Natural History, 1500–1700,” in *The Power of Images in Early Modern Science*, ed. Wolfgang Lefèvre, Jürgen Renn, and Urs Schoepflin (Basel: Birkhäuser, 2003), 141–66.

xii British Library, Sloane MS 1357, f.1r.

xiii British Library, Sloane MS 1346. Henry Power’s move to Wakefield was recorded in his memorandum, cf. British Library, Sloane MS 1358, f. 1.

xiv Henry does not mention his private library in his will. See J.W. Clay, “Dr. Henry Power of New Hall, F.R.S.,” *Papers, Reports, &c., Read before the Halifax Antiquarian Society.*, February 6, 1917, 1–31.

xv British Library, Sloane MS 4019, f. 149-154.

xvi For studies of how catalogues were used in the sale of private libraries, see Giles Mandelbrote, “Sloane’s Purchases at the Sale of Robert Hooke’s Library,” in *Libraries within the Library: The Origins of the British Library’s Printed Collections*, ed. Giles Mandelbrote and Barry Taylor (London: British Library, 2009), 98– 145; Alison Walker, “Sir Hans Sloane’s Printed Books in the British Library,” in *Libraries within the Library: The Origins of the British Library’s Printed Collections*, ed. Giles Mandelbrote and Barry Taylor (London: British Library, 2009), 89–97.

xvii Anthony Grafton, “The History of Ideas: Precept and Practice, 1950-2000 and Beyond,” *Journal of the History of Ideas* 67, no. 1 (2006): 28.

**xviii:** Jacqueline Pearson, “Women Reading, Reading Women,” in *Women and Literature in Britain, 1500–1700*, ed. Helen Wilcox (Cambridge: Cambridge University Press, 1996), 80–99; Leah Knight, Micheline White, and Elizabeth Sauer, *Women’s Bookscapes in Early Modern Britain: Reading, Ownership, Circulation* (Ann Arbor, MI: University of Michigan Press, 2018); Valerie Wayne, Tiffany Stern, and Hannah Leah Crummé, eds., *Women’s Labour and the History of the Book in Early Modern England* (The Arden Shakespeare, 2020).

xix British Library, Sloane MS 1346, f. 2r, under category “English Bookes in fol:”

xx “Min. 68: Jo: Theodori de Bry, florilegium. Oppenheim. 1612 in f. Painted over by Mrs Power.” Sloane 3972C, IV, f. 16v, and “Min. 155. Theodory de Bry Florilegium, Oppenheim 1612. Colour’d by Mrs Power. Fo.” Sloane 3972C, VI, 2498. In *MS 3972C Vol VI Sloane’s catalogue of books and printed ephemera.* Digital Edition. Alexandra Ortolja-Baird, Julianne Nyhan, Victoria Pickering, Kim Sloan and Martha Fleming Eds. 2019. https://enlightenmentarchitectures.reconstructingsloane.org/catalogueMS3972Cvol6/index.html, accessed 3 Oct 2021.

xxi Johann Theodor de Bry, *Florilegium novum, hoc est: variorum maximeque rariorum florum ac plantarum singularium unà cum suis radicibus & cepis, eicones diligenter ære sculptæ*. (Frankfurt, 1612). British Library, 442.g.11.

xxii British Library, Sloane MS 4019, f. 150.

**xxiii:** Matthias de L’Obel, *Icones stirpium, seu Plantarum tam exoticarum quam indigenarum, in gratiam rei herbariæ studiosorum in duas partes digestæ cum septem linguarum indicibus, etc*. (Antverpiæ: Ex officina Plantiniana, 1591). British Library, 443.a.5. For the modified pagination, see p. 225.

xxiv de Bry, British Library, 442.g.11, unpaginated; Parkinson, *Paradisi*, 78; British Library, 443.a.5, verso of frontispiece; John Gerard and Thomas Johnson, *Herball Or Generall Historie of Plantes* (London: Printed by Adam Islip Joice Norton and Richard Whitakers, 1633), 1157-8.

xxv British Library Add MS 5298. This album of Margery’s is analysed in Kim Sloan, “Sir Hans Sloane’s Pictures: The Science of Connoisseurship or the Art of Collecting?,” *Huntington Library Quarterly* 78, no. 2 (2015): 381–415. We do not know whether Margery and Henry grew tulips in their own garden. Tulips were luxurious and expensive plants in this period, as attested by the ‘Tulipmania’ of Holland earlier in the century, cf. Anne Goldgar, *Tulipmania: Money, Honor, and Knowledge in the Dutch Golden Age* (University of Chicago Press, 2008). Yet, we do know that tulips were grown in pots in elite gardens making it at least possible that the Power’s did so, too. Cf. Vera Keller, “A ‘Wild Swing to Phantsy’: The Philosophical Gardener and Emergent Experimental Philosophy in the Seventeenth-Century Atlantic World,” *Isis* 112, no. 3 (2021): 507–30.

xxvi Sloane 3972C, VI, 2498. In *MS 3972C Vol VI Sloane’s catalogue of books and printed ephemera.* Digital Edition. Alexandra Ortolja-Baird, Julianne Nyhan, Victoria Pickering, Kim Sloan and Martha Fleming Eds. 2019. https://enlightenmentarchitectures.reconstructingsloane.org/catalogueMS3972Cvol6/index.html, accessed 3 Dec 2021.

**xxvii:** Odoardo Fialetti, *The Whole Art of Drawing, Painting, Limning, and Etching*, trans. Alexander Browne (London: Peter Stint and Simon Miller, 1660), p.6. William Salmon, *Polygraphice; or The Art of Drawing, Engraving, Etching, Limning, Painting, Washing, Varnishing, Colouring, and Dying* (London: Richard Jones, 1672), p.41.We thank Heather Wolfe for pointing out the connection between Venice paper and tracing techniques.

**xxviii:** British Library, Sloane MS 1357, ff. 88v, 89r.

xxix British Museum, 1975,U.1589, “Ellen Power | British Museum,” accessed November 22, 2021, https://www.britishmuseum.org/collection/term/BIOG42540. We are grateful to Dániel Margócsy for guiding us towards Ellen Power and her sources.

xxx “Min. 104. -273. x Several flowers fruits, Birds & Insects.ainted in water colours done by Mrs. Ellen Power in an Oblong. 4to. It begins wth ye Demask Rose, & ends with the Robin red-breast: should contain 37 leaves, but wants leaf 11th. As the end is a loosi leaf of ye figures of Solid Bodies from the Globa to the Cube.” In *Sloane 3972B offolios listing manuscript material removed from 3972C.* Digital Edition. Alexandra Ortolja-Baird, Julianne Nyhan, Victoria Pickering, Kim Sloan and Martha Fleming Eds. 2019. https://enlightenmentarchitectures.reconstructingsloane.org/catalogueMS3972B/index.html, accessed 3 Oct 2021. On Ellen Power, see Kim Sloan, *A Noble Art: Amateur Artists and Drawing Masters, c.1600-1800* (London: British Museum Press, published for the Trustees of the British Museum by British Museum Press, 2000), 60, 86.

xxxi British Museum, 1952,0522. Images were after Jacques Le Moyne de Morgues, *La clef des champs pour trouuer plusieurs animaux, tant bestes qu*’*Oyseaux, auec plusieurs fleurs & fruitz* (Londre: Imprimé aux Blackefriers, T. Vautrollier pour Jaques le Moyne, dit de Morgues paintre, 1586).

**xxxii:** British Museum, 1962,0714.1. For more on these watercolours, see Monique Kornell and Dániel Margócsy, “Jacques Le Moyne de Morgues (ca. 1533-1588): Picturing Plants in the 16^th^ Century,” forthcoming.

**xxxiii:** Adriaen Collaert, *Avium vivæ icones in æs incisæ et editæ ab A. Collardo* (Antwerp, 1610).

**xxxiv:** For example, Embroidered picture, seventeenth-century England, Cooper Hewitt, 18616355.

xxxv British Library, Sloane MS 1357, f. 70r.

**xxxvi:** For Henry’s interest in the histories of scientific disciplines, see Dmitri Levitin, *Ancient Wisdom in the Age of the New Science: Histories of Philosophy in England, c. 1640-1700* (Cambridge University Press, 2015), 8183.

**xxxvii:** British Library, Sloane MS 1343, f. 58r.

**xxxviii:** Simon Werrett, *Thrifty Science: Making the Most of Materials in the History of Experiment* (Chicago: The University of Chicago Press, 2019), 121-2.

**xxxix:** Walter Charleton and Pierre Gassendi, *Physiologia Epicuro-Gassendo-Charltoniana: Or A Fabrick of Science Natural, upon the Hypothesis of Atoms* (London: Thomas Newcomb, 1654), 116. Robert Hooke, *Micrographia* (London: John Martyn, 1665), 228. We thank Heather Wolfe for bringing the use of Venice paper in microscopy to our attention.

xl Henry Power, *Experimental Philosophy, in Three Books* (London, England, 1664), 54, 82.

xli Sloan, *Sir Hans Sloane’s Pictures*, 405.

xlii *Experimental Philosophy*, 50.

**xliii:** British Library, Sloane MS 1343, f. 80v.

xliv *Experimental Philosophy*, 48-52. For more on Henry’s observation of poppy seeds, see Christoffer Basse Eriksen, “Picturing Seeds of Poppies: Microscopes, Specimens, and Representation in Seventeenth-Century English Botany,” *Nuncius* 37, no. 2 (2022): 1–28.

xlv Jennifer Stead, “Dr Henry Power and his Alterations at New Hall, Elland 1656-1664”, *Old West Riding: A Collection of Original Articles* 8, nr. 2 (1988): 8–17.

xlvi Stead, 9–10. In April 1658, Henry noted that on “16 April 1658 my wife payd the Gardiner at new Hall 30s & 1s out plus for his payments at N. Hall,” cf. British Library, Sloane MS 1356, f. 54v.

**xlvii:** British Library, Sloane MS 1356, f. 38, cf. Stead, 11.

**xlviii:** Leong, *Recipes and Everyday Knowledge*; Wendy Wall, *Recipes for Thought: Knowledge and Taste in the Early Modern English Kitchen* (Philadelphia: University of Pennsylvania Press, 2016); Simon Werrett, “Food, Thrift, and Experiment in Early Modern England,” *Global Food History* (2021): 1–17.

xlix Henry Power, *Experimental Philosophy* (London: T. Roycroft, 1664).

l Juliet Fleming, *Graffiti and the Writing Arts of Early Modern England* (London: Reaktion, 2001); Katherine Acheson, *Early Modern English Marginalia* (Routledge, 2018).

li Lucy Peltz, *Facing the Text: Extra-Illustration, Print Culture, and Society in Britain, 1769-1840* (San Marino, California: Huntington Library, 2017) remains the most important work on the history of extra-illustration. For the practice of Margery’s contemporary botanists, see Xinyi Wen, *Extra-Illustrating Natural History in Early Modern England*, in progress.

lii Werrett, *Thrifty Science*, 26-30.

liii Examples of annotations of this kind can be found in John Edgington, “Annotations in Copies of Thomas Johnson’s Mercurius Botanicus (1634) and Mercurii Botanici, Pars Altera (1641): Authorship and Provenance,” *Archives of Natural History* 43, no. 2 (2016): 208–20.

liv L’Obel, *Icones stirpium,* 206, 280. British Library, 443.a.5.

lv British Library, Sloane MS 1343.

lvi L’Obel, *Icones stirpium,* 92. British Library, 443.a.5.

lvii Power, *Experimental Philosophy*, 72.

**lviii:** Power, *Experimental Philosophy*, 59. For a discussion of the importance of the colour of blood in seventeenth-century anatomy, see Domenico Bertoloni Meli, “The Color of Blood: Between Sensory Experience and Epistemic Significance,” in *Histories of Scientific Observation*, ed. Lorraine Daston and Elizabeth Lunbeck (Chicago: University of Chicago Press, 2011), 117–34.

lix Due to what is now known as chromatic aberration, the image seen in compound microscopes like the one used by Power would have had distorted colours. For discussion of chromatic aberration and seventeenth-century microscopes, see Edward G. Ruestow, *The Microscope in the Dutch Republic: The Shaping of Discovery* (Cambridge: Cambridge University Press, 1996), 18–19.

lx Power, *Experimental Philosophy*, 13.

lxi Valentina Pugliano, “Ulisse Aldrovandi’s Color Sensibility: Natural History, Language and the Lay Color Practices of Renaissance Virtuosi,” *Early Science and Medicine* 20, no. 4–6 (December 7, 2015): 358–96.

lxii Richard Waller, “A Catalogue of Simple and Mixt Colours, with a Specimen of Each Colour Prefix to its Proper Name”, *Philosophical Transactions of the Royal Society of London* 16 (1686): 28. For discussion, see Elaine Ayers, “Coded Colours: Botanical Histories of Colour Standardization”, *The Site Magazine* 40, no. 2 (2019): 24–39.

**lxiii:** Tawrin Baker, “Color and Contingency in Robert Boyle’s Works,” *Early Science and Medicine* 20, no. 4–6 (2015): 536–61.

lxiv British Library, 442.g.11, unpaginated; L’Obel, Icones stirpium, 92, back of frontispiece. British Library, 443.a.5.

lxv British Library, Sloane MS 1357, f. 88v.

lxvi For an overview of the production of ink in the early-modern period, see Adrian Johns, “Ink,” in *Materials and Expertise in Early Modern Europe*, ed. Emma C. Spary and Ursula Klein (Chicago: University Of Chicago Press, 2010), 101–24,

**lxvii:** For analyses of the knowledge circulating in such genres of books, see the essays collected in Alisha Rankin, *Secrets and Knowledge in Medicine and Science, 1500–1800* (Routledge, 2016).

**lxviii:** Romana Sammern, “Red, White and Black: Colors of Beauty, Tints of Health and Cosmetic Materials in Early Modern English Art Writing”, *Early Science and Medicine* 20, no. 4–6 (2015): 397–427; Romana Sammern, “Painting upon the Life. Color Knowledge and Color Practice in English Art Writing and Cosmetics Treatises of the Sixteenth- and Seventeenth Centuries”, in *Colour Histories: Science, Art, and Technology in the 17th and 18th Centuries*, red. Magdalena Bushart og Friedrich Steinle (Berlin: De Gruyter, 2015), 179–98.

lxix For the history of these colours, their provenance and their use, see Julia Nurse, “Pigmented Remedies: The Pharmacy of Colour in Early Modern Europe”, *Canadian Bulletin of Medical History* 38, 1 (2021): 93–117.

lxx For this, see Albrecht Dürer og Thomas Jenner, eds., *A Book of dravving, limning, vvashing or colouring of maps and prints, and the art of painting, with the names and mixtures of colours used by the picture-drawers* (London: M. Simmons for Thomas Jenner, 1652); P. L., *The English academy a drawing book, containing variety of examples of the external parts of men, women, and childrens bodies with the shapes of several creatures frequently used amongst heralds, gold-smiths, &c.* (London: H. Lloyd for Dixy Page, 1672).

lxxi René Descartes, *Discourse on Method, Optics, Geometry, and Meteorology* (Hackett Publishing, 2001), 337– 38; Simon Werrett, “Wonders Never Cease: Descartes’s Météores and the Rainbow Fountain,” *The British Journal for the History of Science* 34, no. 2 (June 2001): 129–47.

**lxxii:** Power, *Experimental Philosophy*, 73.

**lxxiii:** Henry spoke of the oils of tartar and vitriol, yet it is unclear what these oils exactly were. He might have referred to a more liquid variety of the substance known as cream of tartar, or potassium bitartrate, which is a byproduct of winemaking.

**lxxiv:** Power, 74–75.

lxxv Power, 75.

**lxxvi:** Robert Boyle, “The Origine of Formes and Qualities,” in *The Works of Robert Boyle*, ed. Michael Hunter and Edward B. Davis, vol. 5, 14 vols. (Abingdon: Routledge, 1999), 281–443.

**lxxvii:** William R. Newman, *Atoms and Alchemy: Chymistry and the Experimental Origins of the Scientific Revolution* (University of Chicago Press, 2006), chap. 7; Alexander Wragge-Morley, “Robert Boyle and the Representation of Imperceptible Entities,” *The British Journal for the History of Science*, November 2017, 1– 24.

**lxxviii:** Power, *Experimental Philosophy*, 58–61. For the long history of this specific kind of observation, see Karin Ekholm, “Pictures and Analogies in the Anatomy of Generation,” in *Reproduction: Antiquity to the Present Day*, ed. Lauren Kassell, Nick Hopwood, and Rebecca Flemming (Cambridge: Cambridge University Press, 2018), 209–24, and for Harvey’s reluctance towards microscopical observation, see Christoffer Basse Eriksen, “Magnifying the First Points of Life: Harvey and Descartes on Generation and Scale,” *History of Science*, 2021.

**lxxix:** Power, *Experimental Philosophy*, 60.

lxxx Robert Hooke, *Micrographia: Or, Some Physiological Descriptions of Minute Bodies Made by Magnifying Glasses, with Observations and Inquiries Thereupon* (London, 1665), 121.

**lxxxi:** Nehemiah Grew, *The Anatomy of Plants: With an Idea of a Philosophical History of Plants. And Several Other Lectures, Read Before the Royal Society* (London: W. Rawlins, 1682), 38.

